# Editorial—The Last Post

**Published:** 1992-12

**Authors:** Paul Goddard


### Editorial — The Last Post

Sad news and hard luck stories abound in times of financial stringency and I am sorry to add the plight of the West of England Medical Journal to the list of woes. With the last issue of the journal we asked whether or not recipients would be willing to pay £20 per annum to receive four quarterly issues of the journal. Unfortunately despite circularising 4,000 doctors we only received 113 responses (79 yes and 34 no). We have waited to see if more replies would be forthcoming but to no avail.

This is therefore the **final** issue of the West of England Medical Journal. It just remains for me to thank all who have been involved in running the journal, including the editorial committee and board. Most of all my thanks are extended to Mr. Mike Wilson, my predecessor as editor who was unsurpassed in persuading people to write for the journal and kept it together for so long, and to the Chairman of the Editorial Board, Professor Farndon, who was tireless in his efforts to keep the journal alive.

My time as editor has definitely expired and I must say that I have enjoyed much of the experience despite the financial problems that have beset the Bristol Med. Chi. Journal and the West of England Medical Journal during the the time that I have been associated with it (since 1985 as assistant editor and from 1988 as publisher).

Not all is gloomy, however. The title "Bristol Medico-Chirurgical Journal" now reverts back to the Bristol Med. Chi. Society who may wish to continue publishing the journal in a reduced format and to a smaller circulation. Dr. Jim Catterall, consultant physician at the Bristol Royal Infirmary, has been proposed as the next editor but the decision rests with the Bristol Med. Chi. Society.

I have one pleasure remaining and that is to commend to you the contents of this present issue. Dr. Julian Kabala, consultant radiologist at the BRI, has worked long and hard on the commissioning and editing of the mini-symposium on advances in Urology. I enjoyed reading this issue and hope you will do likewise.

Paul Goddard

